# Impact of Dietary Habits on Glycemic Control in Type 2 Diabetic Patients Attending a Tertiary Care Hospital

**DOI:** 10.7759/cureus.87680

**Published:** 2025-07-10

**Authors:** Dania Khawar Rahman, Muhammad Irshad Khan, Wardah Ikram, Mohammed Mohsin Raza

**Affiliations:** 1 Department of Acute Medicine, Ealing Hospital, London, GBR; 2 Department of Medicine, Pakistan Institute of Medical Sciences, Islamabad, PAK; 3 Department of Medicine, Khyber Teaching Hospital, Peshawar, PAK; 4 Department of Endocrinology, Royal Infirmary Hospital, Edinburgh, GBR; 5 Department of Acute Medicine, Allama Iqbal Medical College Lahore, Lahore, PAK; 6 Department of Medicine, Shaikh Zayed Medical College and Hospital, Rahim Yar Khan, PAK

**Keywords:** dietary habits, fiber intake, glycemic control, hba1c, pakistan, sugar intake, type 2 diabetes mellitus

## Abstract

Introduction: Poor glycemic control remains a major challenge in managing type 2 diabetes mellitus (T2DM) in Pakistan, increasing the risk of complications. Dietary habits are a key modifiable factor influencing glycemic outcomes, yet evidence specific to the Pakistani population remains limited.

Objective: To evaluate the association between dietary habits and glycemic control among patients with T2DM attending a tertiary care hospital in Khyber Pakhtunkhwa, Pakistan, and to provide culturally and economically relevant insights to inform dietary recommendations.

Methodology: This cross-sectional study was conducted at the Department of Endocrinology, Khyber Teaching Hospital, Peshawar, over 12 months. A total of 177 adults with T2DM were recruited using consecutive non-probability sampling. Dietary patterns were assessed using a culturally adapted, structured Food Frequency Questionnaire, and glycemic control was determined by glycated hemoglobin (HbA1c), with <7% considered good control. Associations between dietary variables and glycemic control were analyzed using Chi-square tests, t-tests/ANOVA, and multivariate logistic regression adjusting for potential confounders.

Results: Among the 177 participants (mean age 54 ± 10.6 years; 55% female), 118 (66.7%) had poor glycemic control. High sugar intake (more than two servings/day) was significantly associated with higher mean HbA1c (8.6 ± 1.7%) compared to low sugar intake (less than one serving/day; 7.5 ± 1.1%, p = 0.011). Inadequate fiber intake (less than five servings/day) was linked to higher HbA1c (8.3 ± 1.5%) than adequate fiber intake (7.4 ± 1.2%, p = 0.004). Participants consuming two or fewer meals/day had the highest HbA1c (8.7 ± 1.5%), compared to those eating three meals/day (8.0 ± 1.4%) or more than three meals/day (7.8 ± 1.3%, p = 0.027). Multivariate analysis confirmed high sugar intake (adjusted odds ratio (AOR) = 2.67; 95% CI: 1.39-5.12), inadequate fiber intake (AOR = 2.45; 95% CI: 1.19-5.02), and low meal frequency (AOR = 2.21; 95% CI: 1.02-4.79) as independent predictors of poor glycemic control, after adjusting for age, gender, BMI, diabetes duration, and treatment regimen.

Conclusion: High sugar consumption, inadequate fiber intake, and infrequent meals were significantly associated with higher HbA1c and poor glycemic control in this cohort of Pakistani T2DM patients. These findings underscore the importance of culturally sensitive dietary counseling and targeted interventions to improve glycemic outcomes in this population.

## Introduction

People with type 2 diabetes mellitus (T2DM) exhibit insulin resistance and progressive pancreatic β-cell dysfunction, which lead to persistent hyperglycemia over time. Globally, T2DM has emerged as a major health concern among adults, with its prevalence expected to rise significantly in the coming decades [1,2]. The increasing burden of diabetes poses critical challenges to healthcare systems due to the associated macrovascular and microvascular complications, such as cardiovascular disease, nephropathy, neuropathy, and retinopathy [3].

Optimal glycemic control is a cornerstone of diabetes management, aimed at preventing both acute and chronic complications [4]. Fasting plasma glucose and glycated hemoglobin (HbA1c) remain the standard indicators for monitoring glycemic status [5]. Achieving good metabolic control depends on a combination of pharmacologic therapy, regular physical activity, and, importantly, healthy dietary modifications [6]. Dietary habits significantly influence glycemic regulation by affecting insulin sensitivity, glucose metabolism, and postprandial glucose excursions [7].

International studies have consistently demonstrated the benefits of consuming whole grains, dietary fiber, fruits, and vegetables, while limiting refined carbohydrates, saturated fats, and added sugars [8]. In Pakistan and South Asia, however, unique dietary customs, such as high consumption of refined wheat-based breads, white rice, sugary tea, and fried foods, are prevalent and culturally ingrained, potentially impeding glycemic control [9,10]. These regional patterns underscore the importance of culturally tailored dietary interventions.

Despite advances in pharmacotherapy, many patients continue to struggle with poor glycemic control, highlighting the need to address modifiable lifestyle factors, particularly diet, to complement medical treatment and improve outcomes [11]. However, evidence specific to the dietary patterns and their impact on glycemic markers in Pakistani populations remains limited [12]. This study aims to fill this gap by evaluating the association between dietary habits and glycemic control among T2DM patients attending a tertiary care hospital, thereby informing locally relevant dietary recommendations. The objective of this study was to evaluate the association between dietary habits and glycemic control among patients with T2DM attending a tertiary care hospital in Khyber Pakhtunkhwa, Pakistan, and to provide culturally and economically relevant insights to inform dietary recommendations in this population.

## Materials and methods

Study design and setting

Over the course of 12 months, from April 18, 2020, to April 18, 2021, this cross-sectional study was carried out at the Department of Endocrinology, Khyber Teaching Hospital (KTH), Peshawar, a significant tertiary care facility in Khyber Pakhtunkhwa, Pakistan. The purpose of the research was to assess how dietary practices affected the glycemic management of individuals with type 2 diabetes.

Sample size calculation

The sample size was calculated using the standard single population proportion formula: n=Z^2^⋅p(1−p)/d^2^, assuming a 60% prevalence of poor glycemic control (p=0.6 ) based on prior studies [13], a 95% confidence level (Z=1.96), and a 7% margin of error (d=0.07). This yielded a minimum required sample of 171, which was increased by 5% to 177 to account for potential non-response or missing data.

Study population and sampling

Participants had to be 18 years of age or older, enrolled in the outpatient endocrinology clinic throughout the research period, and have a verified diagnosis of type 2 diabetes mellitus for at least a year. Patients with severe diseases, type 1 diabetes, gestational diabetes, or cognitive impairment that might compromise the gathering of accurate data were not included. Consecutive sampling was used to enroll participants until the target sample size was achieved.

Data collection

After obtaining written informed consent, data were collected through face-to-face interviews using a pre-tested, structured questionnaire (Appendices). Dietary intake was assessed with a semi-quantitative Food Frequency Questionnaire (FFQ) based on the conceptual framework of the Harvard/Willett FFQ [13] and adapted from the validated FFQ developed by Bowen et al. for urban and rural Indian populations [14]. This instrument was selected because of the close cultural and dietary similarities between Indian and Pakistani populations, particularly in staple foods, cooking practices, and meal patterns, making it highly relevant to our setting. The adapted FFQ was pre-tested in a pilot sample of 20 patients to ensure clarity, cultural relevance, and completeness, with minor modifications incorporated before final use.

Glycemic control was assessed using the most recent HbA1c value from hospital laboratory records, ensuring the test was performed within the preceding three months. Additional clinical data, including diabetes duration, current treatment regimen, and the presence of diabetes-related complications, were also documented.

Dietary behaviors were classified according to established recommendations: fiber intake was considered inadequate if less than five servings of fruits and vegetables per day, consistent with WHO guidelines recommending at least 400 grams daily [15]; sugar intake was categorized as high if more than two daily servings of added sugars or sugary beverages, following American Heart Association guidance [16]; and meal frequency was classified as low (two or fewer meals/day), moderate (three meals/day), or high (more than three meals/day), based on evidence linking infrequent meals to poorer metabolic outcomes [17].

Physical activity, medication adherence, and socioeconomic status were collected through simple self-reported questions. Physical activity was assessed by asking participants to describe the type, frequency, and duration of their usual daily activities, which were then categorized as low, moderate, or high based on reported patterns. Medication adherence was evaluated by inquiring whether participants regularly took their prescribed diabetes medications as directed, and whether they missed doses in the past week. Socioeconomic status was determined by collecting information on education level, occupation, and approximate monthly household income, which were used to classify participants into broad socioeconomic categories. These variables were incorporated into the analysis to adjust for potential confounding effects and improve the robustness of the findings.

To enhance accuracy and minimize recall bias, participants were asked about their typical dietary intake over the previous three months, with standardized probing for portion sizes and frequencies. Quality control procedures included daily review of completed questionnaires to identify and correct missing or inconsistent responses.

Data analysis

SPSS version 26 (IBM Corp., Armonk, NY, USA) was used for data entry and analysis. The research population's characteristics and eating habits were summed up using descriptive statistics, which include means, standard deviations, frequencies, and percentages. Using independent t-tests or ANOVA for continuous variables and chi-square tests for categorical data, associations between dietary practices and glycemic control were evaluated. To find independent dietary predictors of poor glycemic control while adjusting for variables such as age, gender, body mass index (BMI), and length of diabetes, multivariate logistic regression was used. To identify independent dietary predictors of poor glycemic control, multivariate logistic regression analysis was performed, adjusting for potential confounders including age, gender, duration of diabetes, BMI, medication regimen (insulin vs. oral agents), and presence of diabetes-related complications. Medication adherence, physical activity, and socioeconomic status were not measured and therefore could not be adjusted for in this analysis. P-values below 0.05 were regarded as statistically significant.

Ethical considerations

The hospital's institutional ethics committee examined and approved the research protocol (approval 892/DI/KMC). Prior to enrollment, all individuals provided written informed consent. Throughout the trial, patient data were kept anonymous and confidential. The fact that participation was entirely voluntary and that withdrawal at any moment would not affect medical treatment was explained to the participants.

## Results

A total of 177 patients with type 2 diabetes mellitus participated in the study, with a mean age of 54 years and a predominance of females. The average disease duration exceeded eight years, and most participants were overweight based on BMI. Glycemic control was generally suboptimal, with a mean HbA1c above recommended targets and only about one-third achieving good control (HbA1c < 7%). Regarding behavioral factors, the majority reported consuming three meals per day, while fewer adhered to recommended fiber intake, limited sugar intake, or adequate physical activity. Nearly half of the patients reported inadequate medication adherence, and a substantial proportion belonged to lower socioeconomic strata, underscoring potential barriers to optimal diabetes management (Table 1).

**Table 1:** Socio-Demographic and Clinical Characteristics of Participants (n = 177). BMI: Body Mass Index. HbA1c: Glycated Hemoglobin. Data presented as mean ± standard deviation (SD) or frequency (%). Good glycemic control: HbA1c < 7%; Poor glycemic control: HbA1c ≥ 7%. Meal frequency: number of main meals consumed daily. Sugar intake: servings of added sugars or sugar-sweetened beverages per day. Fiber intake: servings of fruits and vegetables per day, with WHO recommending five or more servings/day. High fat intake: frequent consumption of fried and processed foods. Socioeconomic status: self-reported income level categorized as low, middle, or high. Medication adherence: as self-reported by patients. Physical activity: self-reported regular engagement in ≥ 150 minutes/week of moderate activity considered adequate.

Variable	Category/Value	n (%) / Mean ± SD
Age (years)	—	54.2 ± 10.6 (range 30–75)
Gender	Male	79 (44.6%)
	Female	98 (55.4%)
Duration of Diabetes (years)	—	8.4 ± 4.9
BMI (kg/m²)	—	27.8 ± 3.5
Glycemic Control	Good (HbA1c < 7%)	59 (33.3%)
	Poor (HbA1c ≥ 7%)	118 (66.7%)
Meal Frequency	≤ 2 meals/day	23 (13.0%)
	3 meals/day	106 (59.9%)
	> 3 meals/day	48 (27.1%)
Sugar Intake	Low (<1 serving/day)	65 (36.7%)
	Moderate (1–2 servings/day)	71 (40.1%)
	High (>2 servings/day)	41 (23.2%)
Fiber Intake	Adequate (≥ 5 servings/day)	44 (24.9%)
	Inadequate (<5)	133 (75.1%)
Fat Intake	High	69 (39.0%)
	Not high	108 (61.0%)
Socioeconomic Status	Low	65 (36.7%)
	Middle	85 (48.0%)
	High	27 (15.3%)
Medication Adherence	Adequate	96 (54.2%)
	Inadequate	81 (45.8%)
Physical Activity	Adequate	72 (40.7%)
	Inadequate	105 (59.3%)

**Figure 1:**
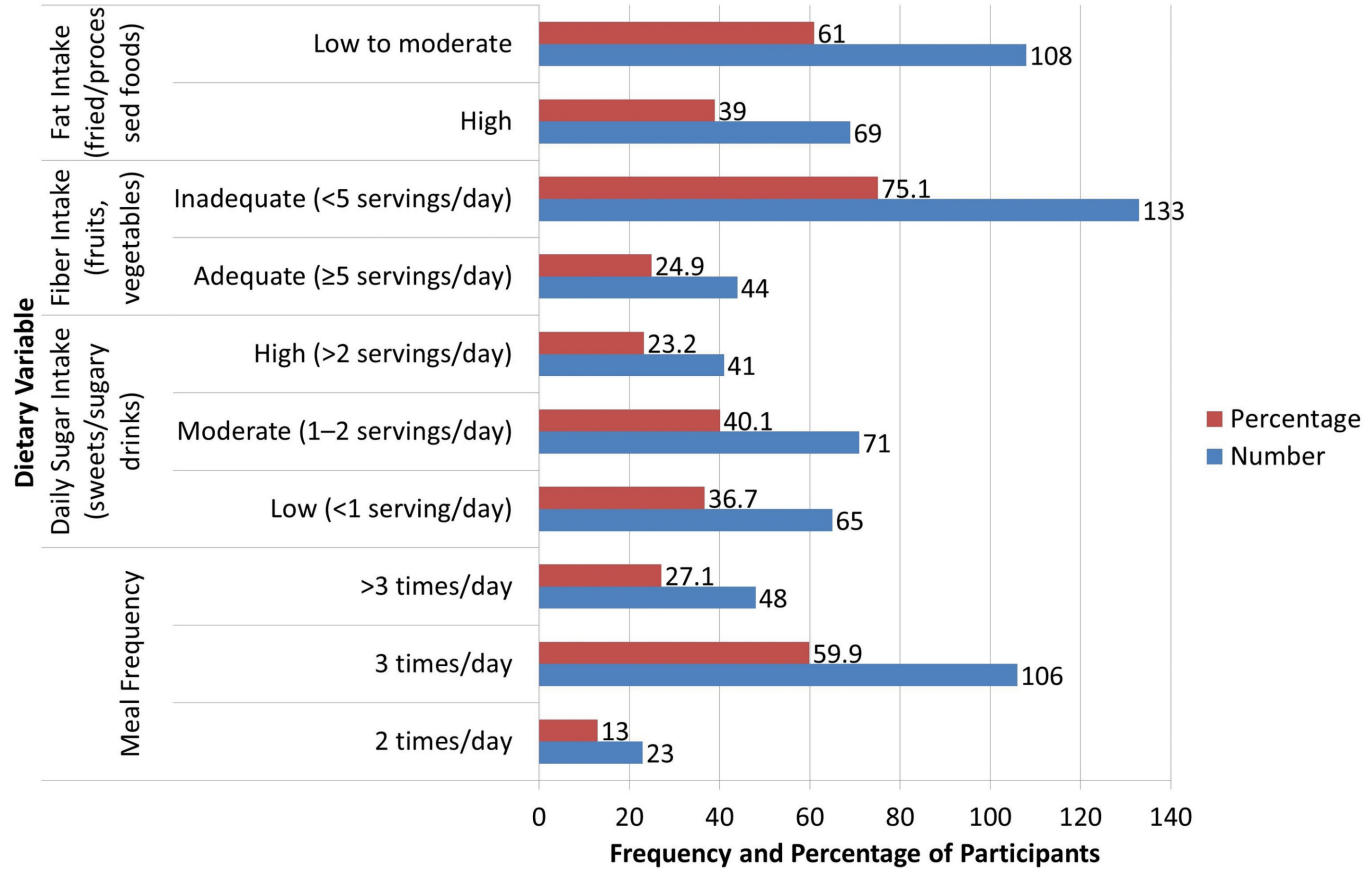
Dietary Habits of Study Participants (n = 177). Dietary behaviors categorized per WHO assessed using the Food Frequency Questionnaire (FFQ). Bold addition: Dietary habits were further analyzed adjusting for potential confounders including age, gender, BMI, diabetes duration, treatment regimen, and complications.

Regarding dietary patterns, as illustrated in Figure 1, the majority of patients (106, 59.9%) reported consuming three meals per day, followed by 48 (27.1%) patients who consumed more than three meals, and 23 (13.0%) who consumed only two meals daily. Daily sugar intake was moderate (one to two servings) in 40.1%, high (two or more) servings in 23.2%, and low (fewer than one serving) in 36.7%. Fiber intake, including fruits and vegetables, was found to be inadequate in 133 (75.1%) patients, while only 44 (24.9%) met the recommended five servings per day. Fat intake, measured based on consumption of fried and processed foods, was high in 69 (39%) patients. These findings indicate a high prevalence of unhealthy dietary habits among the study population.

The Chi-square analysis (Table 2) revealed significant associations between dietary habits and glycemic control. A statistically significant association was observed between meal frequency and glycemic control (χ² = 6.39, p = 0.041), with better control seen among individuals consuming more than three meals per day (25 [52.1%] had good control) compared to those eating only twice daily (three [13.0%] had good control). Sugar intake also showed a significant relationship with glycemic status (χ² = 8.73, p = 0.013), where individuals with low sugar intake (fewer than one serving/day) had better glycemic control (33 [50.8%]) than those with high sugar intake (more than two servings/day) (six [14.6%]). Additionally, fiber intake demonstrated a strong association (χ² = 7.58, p = 0.006), with 33 (75.0%) participants who had adequate fiber intake achieving good glycemic control compared to only 26 (25.6%) among those with inadequate intake.

**Table 2:** Association Between Dietary Habits and Glycemic Control (Chi-square Test). *P-values less than 0.05 were significant.

Dietary Variable	Group	Poor Control n (%)	Good Control n (%)	χ² Value	p-value
Meal Frequency	2 times/day	20 (87.0%)	3 (13.0%)	6.39	0.041*
	3 times/day	75 (70.8%)	31 (29.2%)		
	>3 times/day	23 (47.9%)	25 (52.1%)		
Sugar Intake	Low (<1 serving)	30 (49.2%)	31 (50.8%)	8.73	0.013*
	Moderate (1–2)	56 (79.0%)	15 (21.0%)		
	High (>2 servings)	32 (85.4%)	6 (14.6%)		
Fiber Intake	Adequate	11 (25.0%)	33 (75.0%)	7.58	0.006*
	Inadequate	99 (74.4%)	34 (25.6%)		

Statistically significant differences were observed when comparing mean HbA1c levels across different dietary behaviors using ANOVA and t-tests. Participants consuming only two meals per day had the highest average HbA1c level (8.7 ± 1.5), followed by those eating three meals (8.0 ± 1.4), and those consuming more than three meals per day (7.8 ± 1.3) (p = 0.027). Individuals with high sugar intake exhibited significantly higher HbA1c levels (8.6 ± 1.7) compared to those with low sugar intake (7.5 ± 1.1; p = 0.011). Similarly, patients with adequate fiber intake demonstrated better glycemic control (7.4 ± 1.2) than those with inadequate fiber consumption (8.3 ± 1.5; p = 0.004). These findings suggest that meal frequency, sugar intake, and fiber consumption have a direct and measurable impact on HbA1c levels in patients with type 2 diabetes (as shown in Table 3).

**Table 3:** Comparison of Glycated Hemoglobin (HbA1c) Levels Across Dietary Patterns (ANOVA and t-tests). *P-values less than 0.05 were significant.

Dietary Variable	Group	Mean HbA1c ± SD	p-value	F-value
Meal Frequency	2 times/day	8.7 ± 1.5	0.027*	3.65
	3 times/day	8.0 ± 1.4		
	>3 times/day	7.8 ± 1.3		
Sugar Intake	Low	7.5 ± 1.1	0.011*	4.65
	Moderate	8.2 ± 1.3		
	High	8.6 ± 1.7		
Fiber Intake	Adequate	7.4 ± 1.2	0.004*	8.32
	Inadequate	8.3 ± 1.5		

In multivariate logistic regression analysis, several dietary behaviors emerged as significant independent predictors of poor glycemic control. Patients with high sugar intake had nearly three times higher odds of poor control (adjusted odds ratio (AOR): 2.67; 95% CI: 1.39-5.12; p = 0.003), while those with inadequate fiber intake had more than double the odds (AOR: 2.45; 95% CI: 1.19-5.02; p = 0.015). Similarly, consuming only two meals per day significantly increased the odds of poor control (AOR: 2.21; 95% CI: 1.02-4.79; p = 0.043), and a diabetes duration longer than 10 years was also associated with higher odds (AOR: 1.89; 95% CI: 1.01-3.52; p = 0.046). Other factors, including age over 55 years, female gender, BMI >25 kg/m², and high fat intake, were not statistically significant predictors in this model. However, their effect estimates and confidence intervals are reported in Table 4. Multivariate logistic regression analyses adjusted for potential confounders including age, gender, BMI, duration of diabetes, treatment regimen, and complications confirmed that high sugar intake, inadequate fiber intake, and low meal frequency remained independently associated with poor glycemic control.

**Table 4:** Multivariate Logistic Regression for Predictors of Poor Glycemic Control. *P-values less than 0.05 were significant. Odds ratios (OR) adjusted for age, gender, BMI, duration of diabetes, treatment regimen (insulin vs. oral agents), and presence of diabetes-related complications.

Variable	Adjusted OR	95% CI	p-value
Age (>55 years)	1.42	0.78–2.59	0.251
Female Gender	1.17	0.65–2.12	0.591
BMI (>25 kg/m²)	1.29	0.70–2.38	0.412
Duration of Diabetes (>10 yrs)	1.89	1.01–3.52	0.046*
High Sugar Intake	2.67	1.39–5.12	0.003*
Inadequate Fiber Intake	2.45	1.19–5.02	0.015*
High Fat Intake	1.36	0.74–2.51	0.316
Low Meal Frequency (2/day)	2.21	1.02–4.79	0.043*

## Discussion

This study demonstrated a strong association between dietary habits and glycemic control in patients with type 2 diabetes mellitus. The majority of patients had poor glycemic control, as indicated by an average HbA1c of 8.1%. High sugar intake, low fiber consumption, and reduced meal frequency (specifically two meals per day) were significantly associated with elevated HbA1c levels and increased risk of poor glycemic control. Statistical analyses confirmed that these dietary factors remained significant even after adjusting for confounders such as age, gender, and diabetes duration. These findings reinforce the critical role of diet in managing type 2 diabetes.

The results align with prior studies that emphasize the detrimental impact of excessive sugar consumption on blood glucose regulation. High sugar intake, particularly from sugary beverages and processed foods, has been consistently linked with increased insulin resistance and worsened glycemic markers [18]. Similarly, adequate intake of dietary fiber, especially from fruits and vegetables, has been shown to delay gastric emptying, reduce postprandial glucose spikes, and improve insulin sensitivity [19]. Our study’s finding that individuals with low fiber intake had significantly higher HbA1c supports these previously established mechanisms.

In terms of meal frequency, the current findings indicate that consuming only two meals per day is associated with poorer glycemic outcomes. Research supports the idea that more frequent, smaller meals help regulate glucose metabolism by preventing large postprandial glucose excursions [20,21]. Skipping meals has been correlated with increased hunger, overeating, and poor metabolic regulation, which may explain the elevated HbA1c observed in the low-frequency meal group [22]. Furthermore, while fat intake showed a trend toward association with glycemic control, it did not reach statistical significance in this study. This may be due to heterogeneity in the types of dietary fat consumed (e.g., varying proportions of saturated and unsaturated fats), potential underreporting or misclassification in self-reported dietary data, and the limited sample size reducing the power to detect significant associations.

Compared to previous studies from both high- and low-resource settings, our findings align with the global understanding that diet quality, particularly fiber-rich, low-sugar, balanced meals, plays a crucial role in glycemic regulation [23,24]. By confirming these associations in a real-world, culturally specific Pakistani cohort, our results contribute regionally relevant evidence to the robust global literature demonstrating that lifestyle interventions complement pharmacotherapy in optimizing glycemic control. While we used a standardized, culturally adapted FFQ to assess dietary intake, it remains a subjective measure relying on self-report rather than an objective biomarker-based assessment. The high prevalence of poor dietary patterns in our Pakistani cohort reflects regional challenges, including traditional diets dominated by refined carbohydrates (e.g., white rice, naan) and deep-fried foods, coupled with low consumption of fresh fruits and vegetables due to limited awareness and ingrained cultural preferences.

The Gross Domestic Product (GDP) per capita in Pakistan was estimated at USD 1,643.68 in 2024, approximately 13% of the global average, reflecting persistent economic constraints [25]. The estimated average monthly per capita income is around PKR 45,800 (about USD 1,600 annually), with a substantial proportion of households living below the poverty line (Pakistan Economic Survey 2023-24) [25,26]. These financial limitations make healthier dietary options - such as whole grains, lean proteins, and fresh produce - less affordable for many families, further emphasizing the need for culturally sensitive and economically feasible dietary interventions in this population [27,28].

Limitations and future suggestions

This study has several limitations that should be considered when interpreting the findings. First, due to its cross-sectional design, it can only demonstrate associations between dietary habits and glycemic control rather than causal relationships. Second, recall and social desirability bias are possible, as dietary intake data were self-reported. Although the FFQ was culturally adapted and pilot-tested for clarity and relevance, it was not formally validated against biomarkers or food diaries, which could improve accuracy in future studies. Finally, as data were collected from a single tertiary care facility, the findings may not be fully generalizable to the broader population of individuals with type 2 diabetes in Pakistan.

To address these limitations, future research should consider multicenter studies conducted across diverse geographic and socioeconomic settings, including rural clinics, public sector hospitals, and private urban centers, to enhance representativeness. Longitudinal or interventional designs that incorporate objective dietary assessments (e.g., dietary biomarkers or food diaries), as well as comprehensive evaluations of physical activity, medication adherence, and socioeconomic status, would offer a more complete understanding of how lifestyle behaviors affect glycemic outcomes. Such approaches would also allow for stronger causal inference and provide more actionable insights for public health interventions.

## Conclusions

This study demonstrates a significant association between dietary habits and glycemic control in individuals with type 2 diabetes mellitus. Participants who consumed fewer meals per day, had higher sugar intake, and reported inadequate fiber consumption were more likely to have poor glycemic control. These dietary behaviors were independently associated with elevated HbA1c levels even after adjusting for confounders such as age, gender, and diabetes duration.

The findings validate globally recognized dietary risk factors for poor glycemic outcomes within the specific sociocultural and economic context of Pakistan, where such evidence has been limited. They underscore the critical role of nutrition in diabetes management, highlighting how decreased meal frequency, insufficient fiber intake, and excessive sugar consumption remain strong predictors of poor glycemic outcomes in this population. These results support the urgent need for culturally tailored patient education and feasible dietary interventions. In resource-limited settings like Pakistan, improving dietary habits may be pivotal in enhancing glycemic control and reducing diabetes-related complications.

## Appendices

**Table 5:** Questionnaire Used in Data Collection. FFQ: Food Frequency Questionnaire Adapted from [13,14]

Section	Variable / Domain	Sub-Variables / Categories	Response Options / Measurement
Sociodemographic Data	Age	Years	
	Gender	—	Male
			Female
	Marital Status	—	Single
			Married
			Widowed
			Divorced
	Education Level	—	No formal education
			Primary
			Secondary
			Higher
	Occupation	—	Employed
			Unemployed
			Retired
	Socioeconomic Status	Monthly household income PKR	low
			middle
			high
	Residence	—	Urban
			Rural
Clinical Characteristics	Duration of Diabetes	Years	
	Family History of Diabetes	—	Yes
			No
	Current Treatment	Type of treatment	Oral agents
			Insulin
			Both
	Medication Regimen Details	Daily doses & frequency	
	Presence of Diabetes-related Complications	Type	Neuropathy
			Retinopathy
			Nephropathy
			Foot ulcers
			None
	Most Recent HbA1c	% (from records, ≤3 months)	
	BMI (kg/m²)	Weight & height measured	
	Blood Pressure (mmHg)	Systolic / Diastolic	
Dietary Behaviors (FFQ-based)	Meal Frequency	—	≤2 meals/day
			3 meals/day
			>3 meals/day
	Sugar Intake	Servings/day of added sugar/sweetened beverages	<1
			1–2
			>2
	Fiber Intake	Servings/day of fruits & vegetables	<5
			≥5
	Fat Intake	Type of fats	High (fried/processed)
			Low (boiled/grilled)
	Snack Consumption	Between meals	Yes
			No
	Breakfast Skipping	—	Yes
			No
Lifestyle Factors	Physical Activity	Frequency	Regular (≥30 min/day)
			Irregular
			None
	Type of Activity	Walking / Exercise / Other	
	Sedentary Behavior	Hours/day sitting	
	Medication Adherence	—	Regular
			Irregular
	Smoking Status	—	Never
			Former
			Current
	Alcohol Consumption	—	Yes
			No
Other Variables	Perceived Stress (self-reported)	—	Low
			Moderate
			High
	Sleep Patterns	Hours/night	<6
			6–8
			>8
	Health Literacy	Ability to read prescription/diet plan	Yes
			No
Quality Control	Interview Completeness	Checked by interviewer	Yes
			No
	Data Source for Clinical Measures	—	Patient record
			Self-reported
